# Understanding non-covalent interactions in larger molecular complexes from first principles

**DOI:** 10.1063/1.5075487

**Published:** 2019-01-03

**Authors:** Yasmine S. Al-Hamdani, Alexandre Tkatchenko

**Affiliations:** Physics and Materials Science Research Unit, University of Luxembourg, L-1511 Luxembourg City, Luxembourg

## Abstract

Non-covalent interactions pervade all matter and play a fundamental role in layered materials, biological systems, and large molecular complexes. Despite this, our accumulated understanding of non-covalent interactions to date has been mainly developed in the tens-of-atoms molecular regime. This falls considerably short of the scales at which we would like to understand energy trends, structural properties, and temperature dependencies in materials where non-covalent interactions have an appreciable role. However, as more reference information is obtained beyond moderately sized molecular systems, our understanding is improving and we stand to gain pertinent insights by tackling more complex systems, such as supramolecular complexes, molecular crystals, and other soft materials. In addition, accurate reference information is needed to provide the drive for extending the predictive power of more efficient workhorse methods, such as density functional approximations that also approximate van der Waals dispersion interactions. In this perspective, we discuss the first-principles approaches that have been used to obtain reference interaction energies for beyond modestly sized molecular complexes. The methods include quantum Monte Carlo, symmetry-adapted perturbation theory, non-canonical coupled cluster theory, and approaches based on the random-phase approximation. By considering the approximations that underpin each method, the most accurate theoretical references for supramolecular complexes and molecular crystals to date are ascertained. With these, we also assess a handful of widely used exchange-correlation functionals in density functional theory. The discussion culminates in a framework for putting into perspective the accuracy of high-level wavefunction-based methods and identifying future challenges.

## I. INTRODUCTION

Across the natural sciences, intermolecular non-covalent interactions manifest in the properties and functions of all matter, from solid state materials to biological systems. Notably among the non-covalent interactions, van der Waals (vdW) dispersion is ubiquitous and its accurate prediction remains one of the more challenging aspects of theoretical modeling. It is the force that enables geckos to stick to walls^1^ and has been proposed as an important force behind the formation of rings around Saturn.^2^ On a smaller scale, non-covalent interactions such as hydrogen bonds shape proteins and their function in biology. The number of atoms in proteins is ordinarily in the thousands, and despite this, the largeness of a system can be a matter of perspective. From the *ab initio* calculation perspective, we consider systems with 20-50 atoms to be modestly sized, whilst molecular systems exceeding 100 atoms are considered large and especially challenging.

**FIG. 1.**
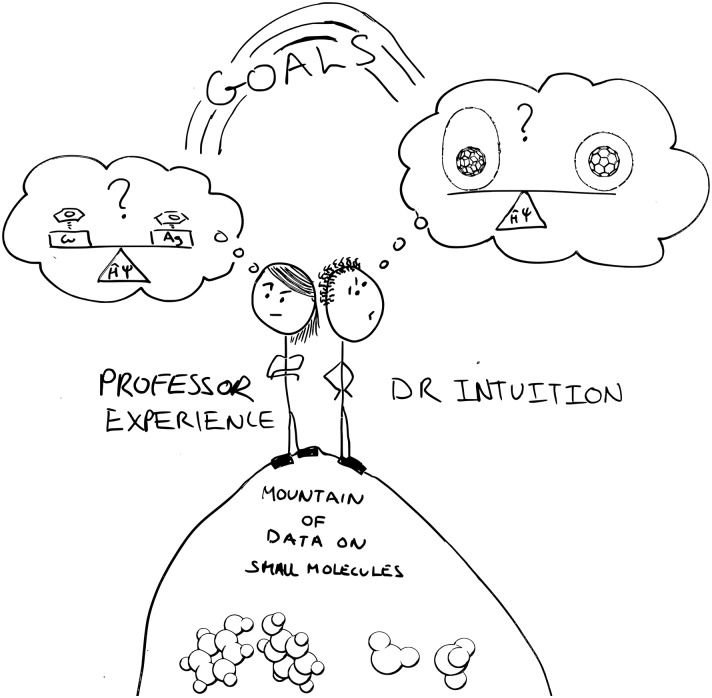
Cartoon illustration of our goal to develop a metric for comparing non-covalent interactions throughout chemical compound space, based on our accumulated knowledge of small molecular systems. Depicted at the bottom is the phenol dimer on the left and the ammonia-water dimer on the right. Top right: the benzene molecule adsorption energy to Cu, Ag, and Au surfaces has been shown to be the same, despite the differences in the materials.^3,4^ Top left: two evidently distinct buckyball-in-a-ring systems have been shown to have the same stability by Hermann *et al.*^5^

Our understanding of non-covalent interactions has been gained from decades of experimental observations supplemented by theoretical predictions, predominantly within the tens-of-atoms regime. This has enabled us to make reasonable hypotheses for the interactions in small systems based only on the knowledge of the molecular geometries. For example, given a pair of molecules like ammonia and water, it is fairly straightforward to guess what configurations will be most energetically favored, based on an understanding of electrostatics and dispersion in small polar molecules. The more ambiguous case of a phenol dimer, for example, is more difficult to predict: we might expect that *π* orbitals may interact via dispersion, whilst the hydroxyl groups will favor a hydrogen bonding orientation. The most stable configuration reflects a combination of these effects, as shown in Fig. 1.

There is a long-standing impetus to exploit the information in the small molecular regime to further our atomistic-level understanding in far more complex systems, such as proteins, for example. In part, this is driven by the concentration of experimental information in the small molecular regime and the greater challenges of studying large and extensive systems under clean ultrahigh vacuum conditions. However, experimental reference information in extensive systems can yield results which are not expected based on our understanding of small molecules only. An example of unprecedented complexity is exhibited by benzene adsorption on coinage metals. It was shown using temperature programmed desorption experiments^6–9^ that the interaction energy of benzene on gold, copper, and silver is very similar—despite the considerable differences in the surface properties.^3,4,10,11^

Another notable example is offered by the experimentally synthesized buckyball-in-a-ring systems of C_70_ hosted by cycloparaphenylenes.^5^ The two configurations shown schematically in Fig. 1 involve *π* − *π* stacking interactions along the length of the ring with the guest buckyball, and it might be guessed that interaction energies will be different and that the structure with the greatest *π* − *π* overlap is the most favorable. Interestingly, the two configurations have almost degenerate interaction energies. Hermann *et al.* demonstrated that a simple dispersion method fails to capture this degeneracy, whereas a many-body formulation can correctly predict the relative energies of these host-guest structures.^5^ Moreover, our understanding of interactions in small molecular systems is not enough to allow us to rationalize such findings *a priori*.

**FIG. 2.**
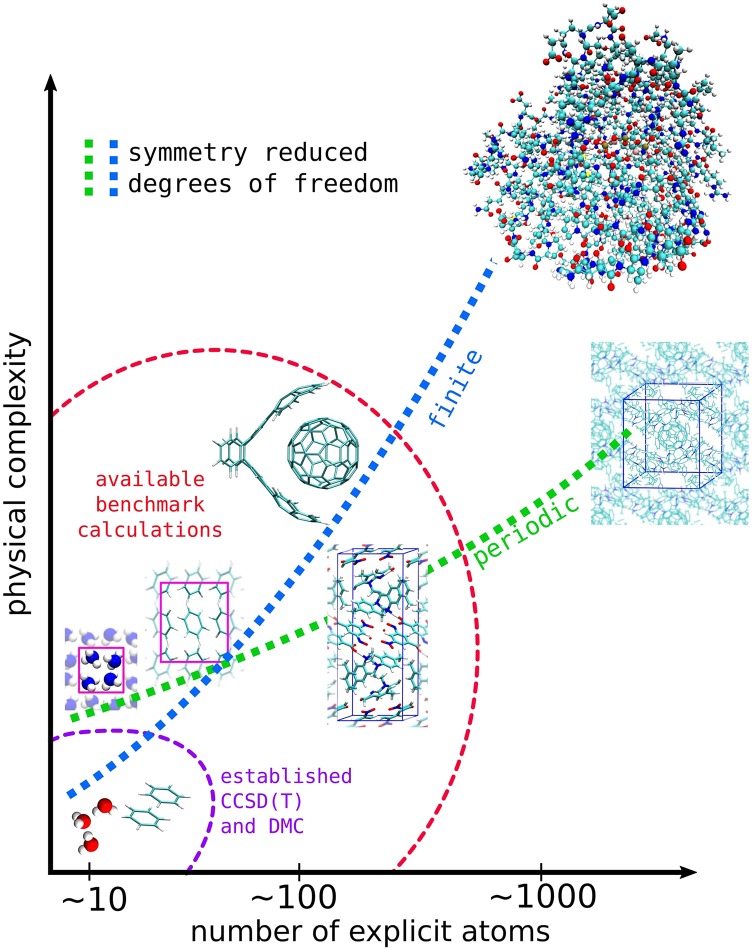
Conceptual overview of the system size and physical complexity of molecular systems. The green and blue dashed lines indicate the computational challenge corresponding to computing periodic and finite molecular systems, respectively. The region enclosed in red dashed line indicates the extent of systems that DMC, localized CCSD(T), and wavefunction based methods in general are being applied to. The smallest systems are well established by now using CCSD(T) and DMC.

Unfortunately, finite temperature and any deviation from pristine experimental conditions have to also be approximated theoretically in order to provide comparable information for computational method development. Unsurprisingly therefore, widely used quantum mechanical methods based on density functional theory (DFT) have been developed predominantly using small molecule benchmarks—where we have the most accurate reference information. For example, Gottschalk *et al.*^12^ recently conducted a double-blind challenge for experiment and theoretical prediction, to determine gas phase furan dimerization preferences at low temperatures. However, simple descriptions of intermolecular interactions in DFT methods, which are useful in predicting small molecules, can fail to comprehensively describe larger systems with more complex interactions. For example, the interaction energy of a water monomer with layered materials, e.g., a semi-conducting carbon nanotube, semi-metallic graphene, and insulating hexagonal boron nitride, is typically overestimated by dispersion inclusive DFT methods.^13–18^ Importantly, shortfalls in DFT approximations are established using higher-accuracy wavefunction based computational methods that have become more practicable in recent years, providing reference information on systems that are particularly challenging to ascertain experimentally. Thus, as more reference information is becoming available for larger systems of 50-200 atoms, we are beginning to gain more understanding of complex long-range intermolecular interactions as well as the theoretical challenges in predicting them. Figure 2 provides a conceptual overview of the physical complexity that accompanies largeness in finite and periodic systems, separately. In general, as the number of symmetry reduced degrees of freedom increases in non-covalently interacting systems, the physical complexity also evolves. Taking Fig. 2 as a road-map, beyond modestly sized systems, such as supramolecular complexes and molecular crystals, lie at the intersection between well-established small molecular systems and macromolecular structures such as proteins. It is therefore timely to assess our capabilities of computing the interaction energies of supramolecular complexes and molecular crystals^19–22^ from high-accuracy wavefunction based methods especially.

In Secs. II–VIII, we discuss the following: we begin with a short account of non-covalent interactions in beyond modestly sized molecular systems in Sec. II. The key aspects of benchmark computational methods are presented in Sec. III. We pay particular attention to the approximations and limitations, which impact on the reliability of predicting intermolecular interaction energies. With those in mind, we report on the most reliable computational predictions to date in supramolecular systems in Sec. IV and molecular crystals in Sec. V. Given the prevalent use of DFT exchange-correlation (*xc*) functionals in first-principles modeling, we also discuss the efforts from DFT studies to match the benchmarks in Sec. VI. Finally, we outline some of the most pertinent challenges that the electronic structure community should tackle in order to make accurate predictions of non-covalent interactions, as well as making suggestions for bridging experiment and computational predictions in Sec. VII.

## II. INTERMOLECULAR INTERACTIONS IN LARGER MOLECULAR SYSTEMS

**FIG. 3.**
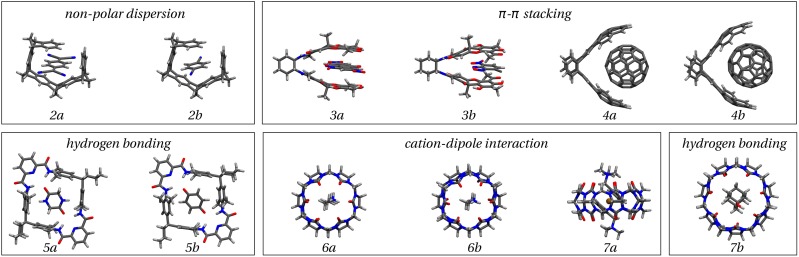
The S12L dataset with groupings made based on the characteristic binding interactions.

Supramolecular complexes are examples of intricate molecular engineering—forming large finite complexes from a few hundred atoms. The buckyball catcher complex (i.e., the host-guest complex shown in Fig. 2 and structure 4a shown in Fig. 3) is a prototypical example of a supramolecular complex, held together with dispersion interactions—predominantly *π* − *π* stacking. In the experiment, such non-covalently bound systems are usually solvated,^23–28^ thus exhibiting similar intermolecular interactions as those present in much larger protein molecules and biological complexes. The challenges of predicting biological ligand systems from quantum mechanics can be read in the review of Ryde and Söderhjelm.^29^ Non-covalent intermolecular interactions can also be important for the application of materials with a long-range structure.^30,31^ In particular, molecular crystals are widespread in pharmaceuticals, explosives, plastics, and organic semiconductors.^32–37^ In all of these applications, the binding strength or cohesion plays a vital role in the corresponding function.

A number of practical challenges have hindered the prediction of beyond modestly sized molecular systems and therefore limited our understanding of them also. For example, non-covalently bound molecular complexes can have many accessible meta-stable energy minima, separated by less than a few kJ/mol.^38,39^ This rich energy landscape requires very accurate predictions or precise measurements to be made experimentally. In addition, computed interaction energies are rarely directly comparable to experimental measurements, necessitating further approximations in order to estimate the effects that separate them. In experiment, sublimation enthalpies are typically measured for molecular crystals at finite temperatures with an estimated accuracy of ∼5 kJ/mol^40^—although the error varies according to the material and typically scales with the magnitude of the sublimation enthalpy. Naturally, zero-point energy contributions and anharmonic effects are included in the experimental observations. Moreover, supramolecular complexes form in solution as opposed to in a vacuum.^23^ Thus, experimental studies for supramolecular structures report association constants which, in addition to the aforementioned thermal effects, incorporate considerable solvent effects. Therefore, non-covalent interactions in systems under the experimental study cannot be easily deduced, whereas theoretical methods complement experiment by providing atomic-level insight. With the combination of experiment and theory over decades, a great deal of understanding has been gained on non-covalent interactions.

The intermolecular interactions that bind molecular complexes extend beyond textbook covalent, ionic, and metallic bonding interactions. There are a number of ways to describe non-covalent interactions,^41^ and a useful classification is based on intermolecular second-order perturbation theory.^42^ That is, electrostatics, induction, dispersion, and exchange-repulsion encompass the four fundamental types of interactions that closed-shell systems exhibit. Note that formally, dispersion is a *type* of vdW interaction, originally explained by London.^41^ There are other vdW interactions, namely, Keesom and Debye, but in this discussion, vdW is invariably used to refer to dispersion unless otherwise stated. Thanks to the widespread importance of non-covalent interactions, other terminology can often be found in biology, chemistry, and physics, but these can be explained in terms of the aforementioned four interactions. For example, hydrogen bonds, *π* − *π* stacking, and ion-ligand interactions are commonly used concepts and each may be comprised of different fundamental types of interactions. The overlap in the terminology also demonstrates that such interactions are intertwined and thus require a balanced self-consistent treatment.^21^ In other words, accurately computing non-covalent interactions require their interdependency to be taken into account. As a result, molecular crystals and supramolecular complexes pose a formidable challenge for theory and experiment,^43^ and predicting intermolecular interactions from first principles has been a long-standing goal of theorists.

For theoretical methods, part of the challenge in predicting non-covalent interactions lies in the expense of computing the correlation energy of electrons, which is inherently a many-body problem. For instance, induction arises from the response of a system to a permanent multipole and requires accurate molecular polarizabilities. The dispersion interaction is also non-trivial to compute, stemming from the Coulomb interaction between the instantaneous correlated fluctuations of electrons, which is present in all electronic systems. The self-consistent nature of intermolecular interactions and the consequent need to go beyond second-order perturbation theory for their reliable description is steadily realised. Here, we mention a few recent studies that highlight the coupling between different types of intermolecular interactions. Furthermore, notable examples can be found in the recent literature.^44–53^ First, using the Drude oscillator model for valence electron fluctuations, it has been shown that the symmetry breaking of the Coulomb potential induced by the confinement of molecules (e.g., within low-dimensional materials) leads to leading-order *repulsive* molecule-pairwise vdW interactions.^54,55^ Given that the confinement of molecules is prevalent, this long-range repulsive term could be relevant to host-guest complexes where the guest molecule is confined in some way by the host. Second, an inhomogeneous electric field induced by a point charge located a few Ångstrom away from the center of mass of a molecular dimer can be used to tailor the vdW dispersion interaction between the molecules, i.e., to enhance or diminish the dispersion interaction depending on the sign of the external charge.^50^ Such findings, taken together, provide strong evidence for the importance of higher-order (beyond second-order) interaction terms for the reliable and predictive description of intermolecular interactions in larger molecules in isolation and embedded in complex polarizable environments. To this end, workhorse methods should be developed on the more complex testing ground of molecular crystals and supramolecular structures, which involve higher-order effects.

Reference information from high-level quantum methods has only become feasible to compute for systems as extensive as molecular crystals and supramolecular complexes in recent years.^5,13,15,16,56,57^ Primarily, this is because methods that can fully treat the correlated nature of electrons are often prohibitively expensive^43,58^ and employ approximations to be practicable for large systems, which increase the uncertainty. Therefore, it is important to assess the status of wavefunction based methods in beyond-modestly sized molecular systems and to consider the accuracy of predicted interaction energies. In Sec. III, we consider the key features of reference methods that have been used to compute larger molecular complexes.

## III. BACKGROUND ON BENCHMARKING WITH FIRST PRINCIPLES METHODS

A reliable modeling of electron correlation necessitates an accurate description of the dynamic response properties of electronic charge density. This is a complex task even for most sophisticated quantum chemistry methods. Most practical electronic-structure approaches either neglect or only partially treat dynamic charge density fluctuations. Benchmark methods typically treat electron correlation explicitly, whereas the workhorse in first principles modeling is DFT, which is an effective mean-field approach. To contextualize the role of more expensive wavefunction based methods, it is helpful to briefly discuss DFT methods. It is well known that whilst DFT is exact, in practice, approximations have to be made due to the unknown *xc* functional. Countless approximate *xc* functionals have been developed over the years, but the crucial sources of error in predicting non-covalent interactions remain the delocalization error and the description of long-range correlation.^59,60^ Correlation energy is often conceptually divided into long-range (∼>7 Å), medium-range (3-7 Å), and short-range (<3 Å), referring to the inter-system separation distances. However, any such division has a certain degree of arbitrariness by construction. In mean-field electronic structure methods, well-established approximations have been implemented for the short-range correlation energy. A greater difficulty lies in approximating the long-range correlation energy, which arises from the Coulomb interaction between well-separated fluctuating charge distributions, also broadly referred to as vdW dispersion.^19,61^

Several *xc* functionals have been developed to treat vdW dispersion interactions to some extent.^62–68^ Such methods are generally referred to as non-local correlation functionals or vdW inclusive functionals. Typically these functionals have been either directly parameterized or later revised to reproduce the reference interaction energies of the S22 and S66 datasets. However, extensive benchmarking across a broad range of systems over the years has demonstrated that vdW inclusive functionals produce a large spread of results,^13,15,65,69–77^ pointing to the need for rigorous benchmarks of overall transferability and accuracy of such approaches.

High-level electronic structure methods can provide important reference information, such as interaction energies and binding configurations, that guide the development of *xc* functionals and classical force-field models and also complement experiments. Such methods include coupled cluster with single, double, and perturbative triple [CCSD(T)] excitations, the random phase approximation (RPA), quantum Monte Carlo (QMC), and symmetry adapted perturbation theory (SAPT). Each of the aforementioned methods accounts for vdW interactions explicitly, albeit approximately. As one might expect, high-level methods can suffer from large memory requirements, poor scaling with system size, and typically large computational prefactors. In addition, each high-level approach comes with its own theoretical limitations. These can manifest in more significant outcomes for molecular crystals and supramolecular systems than for small molecular dimers or solid state systems. An extensive review of the approximations in these methods can be found in the review of Řezáč and Hobza.^78^ For the purposes of this perspective, we consider the relevant approximations, developments, and limitations of these methods more concisely, in the context of molecular crystals and supramolecular complexes. Table I provides an overview of the key attributes of CCSD(T), RPA, SAPT-DFT, and diffusion Monte Carlo (DMC). We also report second-order Møller-Plesset (MP2) theory in Table I since it is a widely used quantum chemical method. However, MP2 lacks screening in its description of long-range correlation and is known to considerably overestimate correlation in dispersion dominated systems. As such, we do not consider it a benchmark method and we do not provide a detailed background aside from including it in Table I. It should be noted that a method known as MP2C has been developed,^79^ which accounts for some screening such that the overestimation of correlation energy is alleviated, improving the general accuracy.^80^

**TABLE I.** Overview of wavefunction based methods that can be used to compute fully *ab initio* predictions.

Method	Interactions		Basis set convergence		Scaling with system size (N)		Comments	
CCSD(T)	•	effectively recovers dynamic correlation and, therefore, dispersion	•	slow and memory scales as N^4^			•	difficult to apply to periodic systems
	•	non-additivity included from screening in dispersion	•	results are often *estimates* in the form of ΔCCSD(T), which is based on a CCSD(T) correction to MP2 energies extrapolated to CBS and is not the same as canonical CCSD(T)^81^	•	N^7^ in the canonical case	•	systems with more than 50 atoms and a converged basis set are not feasible for canonical CCSD(T)
	•	typically based on the HF determinant and therefore lacking static correlation	•	can be improved using explicit correlation factors which give better description of electronic cusps	•	localization and partial correlation, as well as the density-fitting approximation reduce the scaling to near-linear with a large prefactor	•	diffuse functions in the basis set are important to accurately predict non-covalent interactions
MP2	•	some dynamic correlation from uncoupled double excitations derived from 2nd-order perturbation theory yields effectively atom-pairwise dispersion	•	slow and typically requires extrapolation to the CBS limit—similar to CCSD(T)	•	N^5^ in the canonical case	•	strongly overestimates *π* − *π* stacking interactions and not applicable to small-gap systems
	•	no screening at 2nd-order and, thus, cannot account for non-additivity in dispersion	•	basis set convergence can be improved using explicit correlation factors	•	linear scaling can be reached using localization (partial computation of MP2 correlation energy)	•	relies on a cancellation of errors from truncation at 2nd-order and neglect of higher-order terms
			•	plane-wave implementations are available	•	prefactor can be reduced using density-fitting approximation	•	a more costly version, namely, MP2C, uses the difference between uncoupled HF dispersion and coupled KS dispersion to correct MP2 overbinding
RPA	•	Coulomb coupling of direct double excitations up to infinite-order yields dispersion with screening (non-additivity)	•	slow due to the large number of virtually occupied orbitals that are needed	•	N^4^ in most implementations	•	suitable for metals and small-gap systems
	•	standard RPA is missing single excitations (SEs) and second-order screened exchange (SOSEX) terms	•	memory scales as N^4^	•	prefactor can be reduced using density-fitting approximation	•	standard RPA underestimates dispersion dominated interactions, but SE and SOSEX terms can alleviate this
	•	short-range correlation not very accurate in standard RPA					•	results are sensitive to the reference state, but the range-separated *xc* kernel improves short-range correlation
SAPT-DFT	•	dispersion interaction between two molecules computed from 2nd-order *coupled* Kohn-Sham (KS) perturbation theory	•	slow and typically requires extrapolation to the CBS limit	•	N^6^ but can be reduced to N^5^ using density-fitting (which also reduces the prefactor)	•	restricted to computing dimers (although it is theoretically possible to implement for trimers and more)
	•	some screening included in dispersion	•	*ad hoc* scaling factors have been suggested to circumvent the use of large basis sets, which are particularly important for dispersion terms			•	higher-order terms in perturbation theory typically prohibitive to compute
DMC	•	correlation from stochastic sampling of electron configurations yields fully interacting dispersion energy (e.g., screening and non-additivity are accounted for)	•	fast since starting trial wavefunction is used as a guide in importance sampling	•	N^4^ with a large prefactor	•	user intensive method, requiring a careful setup of the trial wavefunction
	•	yields many-electron ground-state solution to imaginary-time Schrödinger equation	•	Jastrow factor (explicit correlation factor) is also used to incorporate cusp conditions	•	computational cost also affected by the size of the target stochastic error, which scales quadratically with the DMC simulation time (e.g., halving the stochastic error in the total energy requires running the calculation for four times longer)	•	fixed-node approximation is a pervasive error
			•	improvements in the basis set should reduce the variance and therefore make the DMC calculation more efficient			•	forces are not typically computed

### A. Coupled cluster theory based methods for large systems

Coupled cluster theory has typically been the method of choice for quantum chemists for attaining the chemical accuracy of 1 kcal/mol in interaction energies. More specifically, CCSD(T) has been dubbed the “gold standard” and widely used to compute reference interaction energies for molecular systems.^82,83^ The winning feature of CCSD(T) is that it effectively recovers dynamic correlation^84^ in a size-consistent manner to the extent that it reproduces experimental reference interaction energies. However, CCSD(T) scales as N^7^, with N being a measure of the system size, and it is not trivially parallelizable. As such, it is computationally prohibitive to obtain canonical CCSD(T) references for molecular crystals and supramolecular systems. A number of approximations and developments in algorithms have extended the use of coupled cluster theory over the years.^85–101^ An approach that has made it feasible to compute supramolecular complexes is the approximation of the CCSD(T) correlation energy via the real space localization of molecular orbitals. This localization enables the number of determinants evaluated in the coupled cluster method to be considerably reduced. Such methods are generally referred to as local CCSD(T). Among these, the domain-based local pair natural orbital (DLPNO) approach has been implemented in conjunction with CCSD(T) and is increasingly used as an alternative to canonical CCSD(T).

Local CCSD(T) methods are typically near-linearly scaling with a relatively large prefactor and can be used to compute interaction energies for systems that are an order of magnitude larger than those typically computed with CCSD(T).^87^ Hence, supramolecular complexes can be computed with local CCSD(T), but such efficiency comes at the cost of reducing the range of correlation energy that is captured. In DLPNO-CCSD(T), weakly interacting electronic states are assumed to contribute insignificantly to the correlation energy and are excluded from the calculation by using a threshold. Discarding a subset of the calculations that collectively yield the correlation energy results in a considerable computational saving. However, weak interactions culminate from states which may be cut off by the threshold, and as such, non-covalently interacting complexes are particularly affected. The threshold for computing the DLPNO-CCSD(T) correlation energy is not based on a real space cutoff, but instead on the overlap amplitudes of natural orbital states. A pre-screening routine is used at the tails of states to indicate their significance. The threshold can be chosen to be as tight as necessary, and in this way, the method can be systematically converged. However, it is important to note that the threshold is system dependent and non-covalently interacting complexes typically require tight thresholds in order to accurately account for dispersion interactions.

Calbo *et al.*^102^ demonstrated in a recent study that supramolecular complexes require tight PNO settings, in order to obtain converged results with DLPNO-CCSD(T). It was shown that normal PNO settings result in DLPNO-CCSD(T) overestimating *π* − *π* and CH-*π* dispersion interactions.^102^ In some cases, however, an *ad hoc* scaling factor is applied to normal PNO obtained results, in order to estimate the converged result. The scaling factor is obtained from small model systems where tight PNO is feasible. This strategy introduces an uncontrolled source of error and relies on extrapolation. Ideally, larger basis sets should be used instead of scaling factors, but due to slow basis set convergence as well as numerical linear dependency problems that arise particularly in *π* systems, it is not always feasible to do so.

Another aspect to consider is the applicability of CCSD(T) methods to periodic systems such as molecular crystals. To date, a few studies have shown that CCSD(T) energies can be computed in a fully periodic system consisting of a few atoms in the unit cell.^103–107^ However, molecular crystals often require tens of atoms in the unit cell in combination with large basis sets, and this has remained impracticable with coupled cluster theory based methods. Alternative approaches to computing energies for periodic systems have been used with coupled cluster methods, and they include fragment based approaches and embedding. The latter can also be used for molecules adsorbed on a surface,^107,108^ whilst the former has mostly been employed for molecular crystals.^109,110^

Fragment approaches take into account different orientations of dimers, trimers, tetramers, and so on within a crystal and a sum over their contributions to the total energy, to yield a lattice energy for the crystal. Note that in taking into account dimers only, the screening from the surrounding molecular environment for any given dimer is not accounted for by such a summation.^111^ In reality, the interaction between the molecules in the crystal is affected by the screening from the molecules (electrons) in the rest of the crystal. This missing component of the energy is sometimes referred to as beyond two-body interactions, and in this particular context, a body refers to a molecule. The fragment approach can be made more accurate by taking into account trimers, tetramers, pentamers, and so on. However, the basis set superposition error (BSSE) in a fragment based approach is non-trivial,^112–115^ and in addition, going to larger fragments quickly becomes unfeasible. Moreover this method is based on the nearsightedness of interacting molecules,^116–118^ and as such, it is less suitable for ionic molecular crystals, for example, where the interactions are longer-ranged.

Ongoing studies are aimed at addressing slow convergences with respect to basis sets, setting physically motivated thresholds, and making periodic calculations feasible. Such developments could eventually enable local CCSD(T) methods to be used in the extensive hundred-atom regime.

### B. SAPT based methods of different flavors

SAPT is a particularly useful method for decomposing the non-covalent interactions into physically meaningful components and, thus, providing physical insight as well as yielding two-molecule interaction energies from a rigorous perturbation theory framework.^41,42^ Since its original formulation using Hartree-Fock (HF) theory, more comprehensive SAPT-DFT has been developed, using coupled KS wavefunctions. In the literature, SAPT-DFT may be referred to as SAPT(DFT)^119^ and DFT-SAPT^120^ owing to development by different groups, but generally both refer to the same method. Using perturbation theory, coupled second-order terms, i.e., dispersion and induction, could in principle be computed exactly with SAPT-DFT. Its exactness relies on knowing the exact *xc* potential in DFT.^121^ In practice, the induction energy tends to be overestimated due to the wrong asymptotic behavior of the approximate *xc* potential. In addition, incorporating asymptotic corrections do not necessarily improve predictions of interaction energies consistently. As it stands, SAPT-DFT scales as N^6^ with system size N, but delivers a significant improvement over vdW-exclusive DFAs for accuracy.

A recent adaptation of the method, XSAPT (short for XPol+SAPT), has been introduced by Lao and Herbert.^122–124^ The authors developed different flavors of XSAPT, all of which share the main feature of using many-body polarized wavefunctions. In this way, the underlying monomer wavefunctions on which SAPT is performed include screening effects from other molecules such that an arbitrary number of molecules can be computed with XSAPT. This is particularly useful for computing supramolecular complexes consisting of more than two molecules, but until a periodic implementation can be developed, molecular crystals remain outside the scope of the method unless a fragment based approach is employed.

SAPT-DFT can also be made more efficient, reducing the scaling to N^5^, by invoking a density-fitting approximation in what is known as DF-DFT-SAPT. The density-fitting approximation has been shown to introduce relatively small sub-kilocalorie errors in the interaction energies of complexes.^120,125,126^ As such, supramolecular complexes can be computed with DF-DFT-SAPT. On the other hand, SAPT-DFT methods are generally strongly basis set dependent, with cubic scaling with respect to the number of basis set functions in the case of DF-DFT-SAPT.^126^ This basis set dependence makes the computation of intermolecular interactions particularly challenging for large systems. Some previous studies^102,127^ have used scaling factors to circumvent the use of a sufficiently large basis set. Applying a basis set scaling factor for larger systems introduces an uncontrolled level of uncertainty in the final result. Nonetheless, unlike other high-level methods, SAPT provides a physically intuitive breakdown of the interactions in supramolecular dimers.

### C. The RPA methods: Building solidly on DFT approximations

The RPA can be derived from many-body perturbation theory or equally from the adiabatic-connection fluctuation-dissipation theorem. An in-depth review of a number of RPA approaches and their derivations is given by Ren *et al.*^128^ In short, the RPA yields the correlation energy, by coupling a system of non-interacting particles to the fully interacting system through adiabatic integration, keeping the density of the two systems equal. Thus, for a given DFT KS density, the RPA correlation energy can be computed and combined with the exact exchange for the system, arriving at a well-founded and more accurate prediction of the energy. In the context of quantum chemistry approaches, the RPA includes the MP2 direct double-excitation correlation energy, but unlike MP2, the RPA also seamlessly accounts for screening since it includes all double-excitation terms up to infinite-order. Screening is considerable in large supramolecular complexes and molecular crystals, and therefore in contrast to MP2, the standard RPA does not overestimate the binding of these systems. Indeed, standard RPA has been shown to underestimate the interaction of dispersion bound materials.^129^ It is known however that two important contributions are not included in the standard formulation of the RPA, namely, the single excitations and the second-order screened exchange (SOSEX) terms. Singles excitations, in particular, have been shown to alleviate the underbinding of the standard RPA^13,129^ and are likely to be important in predicting large molecular complexes also.

Practical factors in the RPA have also been steadily improved over the years.^130,131^ First, the method scales as N^4^ with the system size, which is relatively good compared to the N^7^ scaling of canonical CCSD(T). On the other hand, the RPA methods converge slowly with respect to the number of unoccupied states (which should be rather high) and require large computing memory. In practice, this can necessitate exhaustive convergence tests and extrapolations, amounting to additional computational cost. Nonetheless, a worthy advantage of the RPA method is that it can be readily used to predict all forms of materials, such as finite supramolecular systems and extensive molecular crystals. Moreover, within the robust framework of the adiabatic connection fluctuation-dissipation theorem, we can systematically improve upon RPA.

### D. Quantum Monte Carlo: A stochastic approach to tackling a many-body wavefunction

Quantum Monte Carlo (QMC) is altogether a different approach to quantum chemical methods for solving the Schrödinger equation for a many-electron system. QMC comes in different flavors also, but in the context of weakly interacting realistic systems, it is invariably diffusion Monte Carlo (DMC) that is employed.^132^ DMC is a projector based QMC method that is able to propagate towards the ground-state wavefunction for a system using the imaginary-time Schrödinger equation, given a starting wavefunction with an exact nodal surface. The correlation energy, which includes long-range dispersion, is accounted for in DMC by the stochastic sampling of various electronic configurations around fixed nuclei. A salient feature of QMC methods is their straightforward scaling with the number of computing processors since random walkers in these Monte Carlo based methods can be distributed across thousands of processors. As such, QMC can be performed efficiently on high-performance computers with the possibility of using graphics processing unit (GPU) accelerators.

There are two particularly important approximations that need to be considered in DMC calculations. First, the nodes of the starting wavefunction are fixed in DMC to maintain the Pauli principle of antisymmetry for fermionic wavefunctions. This prevents the DMC solution collapsing to a lower energy bosonic wavefunction. In practice, an approximate nodal surface is used from quantum mechanical calculations, and hence, the fixed node approximation is an important source of error.^133,134^ In the case of non-covalently interacting systems, it has been shown that the nodal surface of the interacting complex is hardly changed from that of the isolated monomers, and therefore, the fixed node error is negligible in the interaction energy.^135^ This has been demonstrated for the S22 dataset, with DMC establishing sub-chemical (<0.01 kcal/mol) accuracy.^135^ As such, it is expected that non-covalently bound molecular crystals and supramolecular structures will also benefit from systematic error cancellation and therefore be less sensitive to the fixed node approximation. In practice, the impact of the fixed node approximation is assessed by computing interaction energies from different trial wavefunctions (i.e., different nodal surfaces) for a given system. Establishing the same interaction energy within the stochastic errors from different trial wavefunctions indicates that the computed property is insensitive to the fixed node approximation.

Second, due to numerical discretization in the DMC algorithm (used for propagating the imaginary-time Schrödinger equation), a time step or lattice spacing^136^ is needed. The method is only exact in the limit of the imaginary time step (or lattice spacing) tending to zero (notwithstanding the fixed node approximation). As such, time step convergence is paramount in DMC calculations to establish the error from the discretization for different systems. Considering the size of large molecular systems, this can be computationally expensive. However, in the recent work of Zen *et al.*, a pervasive size-inconsistency problem stemming from the expression of the branching term in DMC, i.e., the growth/decay term used in propagating random walkers, was explained and addressed.^137^ Size-inconsistency was shown to disappear only for extremely small time steps using the original method, and the error from this is particularly noticeable in interaction energies where the energy is computed with respect to the isolated monomers. The authors introduced a different expression for the branching term, which accounts for the number of electrons in the system as a normalizing constant. In doing so, Zen *et al.* demonstrated that size-consistent total energies can be achieved with an order of magnitude larger time steps.^137^

There are a number of other commonly invoked approximations, such as the use of non-local pseudopotentials in DMC that can affect the accuracy of the electronic structure methods described.^138,139^ In the case of molecular crystals, it is particularly important to consider finite size effects in QMC.^18,140–142^ Overall, DMC requires careful consideration and testing of its biases, especially when computing molecular crystals and supramolecular complexes for which there are few studies to date.

### E. The role of datasets in benchmarking

Datasets are invaluable for developing theoretical frameworks towards benchmark accuracy. Useful datasets for non-covalent interactions include the S22,^143^ S66,^126,144^ BioFragment Database (BFDb),^145^ 3B-69,^146^ C21,^147^ X23,^148^ S12L,^23^ and L7.^58^ Each dataset has a general theme. For example, the S22 and S66 contain modestly sized molecular dimers with less than 50 atoms. BFDb contains biologically relevant complexes with up to 40 atoms, computed at the CCSD(T) level. Dispersion and induction effects at relatively short distances play an important role in these sets. However, the roles of screening and non-additivity in long-range dispersion are not well represented. Given that beyond molecule-pairwise dispersion can play an important role, the 3B-69 dataset reports trimers computed at the CCSD(T) level also. The S12L and L7 datasets present more challenging systems, in which the non-covalent interactions are not well understood, but certainly feature a complex combination of induction, dispersion, and electrostatic interactions. The complexes within L7 and S12L are supramolecular since they contain 50-200 atoms. Predicting these systems with benchmark accuracy is a considerable challenge—as reflected by the scarcity of the reported benchmark values. For the L7 dataset, in particular, the reference interaction energies are given from an *estimated* form of quadratic configuration interaction with singles, doubles, and perturbative triples [i.e., ΔQCISD(T)], using relatively small basis sets. The ΔQCISD(T) interaction energy for the coronene dimer per atom is −0.7 kcal/mol. For comparison, consider that the interaction energies per atom for the parallel-displaced benzene dimer and graphene bilayer are −0.2 and −0.4 kcal/mol, respectively. Whilst it is a possibility that the interaction energy from benzene to graphene is not monotonic, it seems more likely that the coronene dimer interaction energy is overestimated. This could be caused by the underlying use of MP2 in ΔQCISD(T) since MP2 is known to drastically overestimate *π* − *π* dispersion interactions.^149,150^ In the absence of experimental information, such uncertainties in benchmark datasets can only be overcome by the use of more theoretically comprehensive methods. Indeed, more recent DLPNO-CCSD(T) computations of the L7 dataset predict a coronene dimer interaction energy of ∼−19 to −20 kcal/mol or −0.5 kcal/mol per atom.^123,151^ The latter predictions of the coronene dimer interaction energy use explicitly correlated CCSD(T)-F12 and DLPNO-CCSD(T)/CBS and therefore involve less BSSE and reliance on MP2. An advantage of the S12L dataset composed by Grimme^23^ is that back-corrected experimental association constants are available for the complexes. These provide a guide for computational methods, and thus, the S12L dataset has been more widely computed than the L7 dataset.^19,56,72,102,127,146,152^ The S12L dataset, shown in Fig. 3, consists of 12 systems, excluding the small benzene dimer in the original set. Each system exhibits at least one of the following: *π* − *π* interactions, hydrogen bonding, and static polarizable interactions with cations. We consider the most accurate data available for the supramolecular S12L dataset in Sec. IV.

## IV. PREDICTING SUPRAMOLECULAR SYSTEMS

Supramolecular systems consist of hundreds of atoms in a finite, non-periodic arrangement with strong intramolecular interactions, whereas non-covalent interactions are responsible for the formation of the complexes. Due to the sheer size of these systems, these so-called weak interactions amount to large absolute interaction energies in the range of −80 to −550 kJ/mol. In addition, beyond atom-pairwise dispersion has been shown to contribute significantly to the interaction energies, alongside induction and other effects. This poses a phenomenal challenge for computational methods: to predict intricate and finely balanced anisotropic interactions on a large supramolecular scale without having reduced degrees of freedom from periodicity. To date, several methods have been shown to attain sub-chemical accuracy (<0.4 kJ/mol) for small non-covalently bonded systems such as those of the S22 and S66 datasets. However, methods such as CCSD(T), DFT-SAPT, and the RPA require particularly large basis sets to be converged. DMC also may require a significant increase in computer resources since the cost of calculations increases as N^4^, with N being a measure of the system size. Nonetheless, several groups have computed the supramolecular S12L dataset, and to date, this has been done using DMC, ΔDLPNO-CCSD(T), DF-DFT-SAPT, and MP2C. Note that MP2C is essentially MP2 with screening applied to the correlation energy, intended to reduce the overestimation of correlation energy in MP2 theory.^153^

The numerous studies trying to accurately capture non-covalent interactions for systems as fundamental as the water dimer^154^ highlight the formidable challenge the S12L poses. Here, we discuss the most accurate reference calculations undertaken to establish the interaction energies for the S12L dataset. Given the scarcity of reference information for these systems experimentally or theoretically, the reference information is sought from first principles calculations with minimal empiricism or none at all. Three such endeavors have been undertaken for the S12L, and we will review these here, but first, we proceed with some comments on the available back-corrected experimental data.

### A. Binding energies from back-corrected experimental association constants of S12L complexes

Experiments by a number of research groups provide association constants for the supramolecular complexes in the S12L set. Association constants (or binding constants) are straightforwardly related to the Gibbs free energy, bringing the experimentally measurable information closer to computationally predictable quantities. Grimme bridged the gap between the experimental information and theory by using theoretical back-corrections.^23^ More specifically, the rigid rotor harmonic approximation (RRHA) was used to compute the enthalpy at the matching temperature to experiment. In addition, an implicit solvation method, the Conductor-like Screening Model for Realistic Solvents (COSMO-RS) continuum solvation model, is used to approximate the contribution to the association constant from the solvent environment. First, the RRHA neglects any anharmonic contributions which can be expected to be non-trivial for supramolecular systems. Moreover, such systems are not typically rigid and contain low frequency vibrations. The role of low frequency modes has been implicated in anharmonic effects at room temperature, for example, in the binding free energy of DNA base pairs.^155^ Larger and more flexible molecules can be expected to have more low frequency modes, and therefore, it is not clear what impact harmonic approximations have on the binding of such molecules.

Second, the solvation model is expected to have 5%-10% errors^23^ and is less reliable when point charges are present. The latter issue is especially relevant for the cation containing structures 6a, 6b, and 7a of the S12L dataset. The back-corrected interaction energies (Emp-v1), shown in Table II, have been computed at the classical level by taking into account the enthalpic and entropic contributions to the experimental association constants. The original work by Grimme acknowledges the potential sources of error from such methods and reports an estimate of the errors for the experimental association constants as well as the computed back-corrections.^23^ Later Sure and Grimme^156^ made a concerted effort to compare the impact of different solvation models and RRHA results on the computed association constants. In doing so, the back-corrected values were revised to provide the best agreement with the experimental association constants,^156^ referred to as Emp-v2 here. The mean absolute deviation with respect to the experiments for the best-performing empirical method in the study is 2.1 kcal/mol. However, the estimated errors amount to as much as 6 kcal/mol in the interaction energy of some complexes in the S12L. Therefore, Emp-v1 and Emp-v2 serve predominantly as a guideline when comparing predictions of interaction energies.

**TABLE II.** Interaction energies and mean absolute errors (MAEs) for the S12L supramolecule dataset, and units are in kcal/mol. Emp-v1 and Emp-v2 refer to the two versions of empirically back-corrected experimental interaction energies.^23,156^ The estimated errors for Emp-v2 from the work of Grimme^23^ are shown in parentheses; the errors are 10% of the corresponding solvation energy that was computed. The stochastic error in the DMC predictions^56^ is also shown in parentheses. Complexes that have not been computed are noted by n.c. The MAEs are shown with respect to Emp-v1 and Emp-v2. The last rows show MAE and the maximum absolute error (MAX) with respect to Emp-v2 with the estimated errors taken into account. Note that the MAE reported for DMC takes into account the stochastic errors in the DMC results.

S12L Complex	Emp-v1^23^	Emp-v2^156^	DMC^56^	ΔDLPNO-CCSD(T)^127^	DF-DFT-SAPT^157^	MP2C^157^
2a	−29.9	−29.0 (0.9)	−27.2 (0.3)	−30.7	−32.0	−33.5
2b	−20.5	−20.8 (0.4)	−17.2 (1.0)	−23.0	−21.1	−23.0
3a	−24.3	−23.5 (0.5)	n.c.	−23.7	−18.7	−22.9
3b	−20.4	−20.3 (0.3)	n.c.	−23.1	−15.9	−17.2
4a	−27.5	−28.4 (0.6)	−25.8 (1.5)^a^	Unconverged	−36.0	−41.0
4b	−28.7	−29.8 (0.7)	n.c.	Unconverged	−37.1	−41.8
5a	−34.8	−33.4 (0.8)	−33.4 (1.0)	−33.4	−33.8	−37.3
5b	−21.3	−23.3 (0.4)	n.c.	−23.0	−23.1	−25.2
6a	−77.4	−82.2 (6.0)	−81.0 (1.6)	−79.8	−82.6	−84.7
6b	−77.0	−80.1 (6.0)	n.c.	−77.8	−79.1	−81.0
7a	−131.5	n.c.	n.c.	−123.9	−135.0	−139.4
7b	−22.6	−24.2 (0.6)	−24.1 (1.8)	−22.7	−27.0	−28.1
MAE [Emp-v1]		1.5	1.2	2.1	4.0	5.7
MAE [Emp-v2]	1.6		0.9	1.5	2.9	4.4
MAE [Emp-v2 with errors]	0.3		0.6	0.7	2.4	3.6
MAX [Emp-v2 with errors]	1.6		2.2	2.5	7.0	12.0

^a^ A more accurate, revised DMC value is −30.4 kcal/mol, computed by Zen *et al*.^137^

### B. High-level wavefunction based methods for supramolecular complexes

**FIG. 4.**
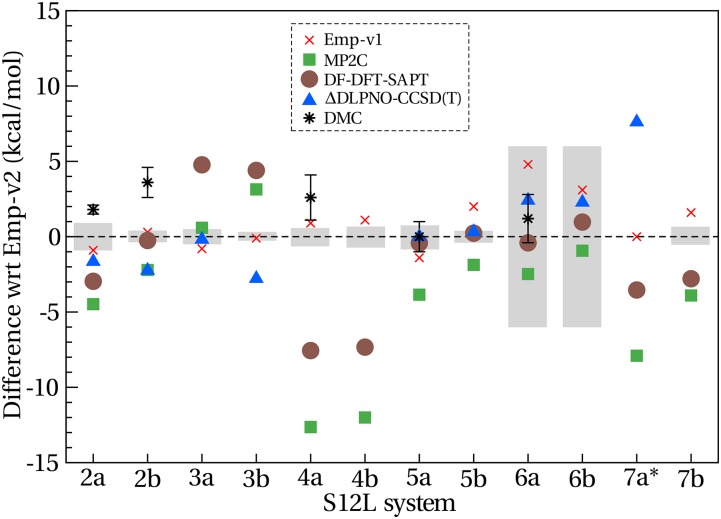
Differences in the interaction energy between a number of high-level wavefunction based methods and the back-corrected experimental values (Emp-v2).^156^ Emp-v1 refers to the original back-corrected data provided by Grimme.^23^ The gray bars indicate the estimated error from empiricism in Emp-v2. There is no revised Emp-v2 data for complex 7a. Data are adapted from studies in the literature.^56,127,157^

Hesselmann and Korona computed DF-DFT-SAPT interaction energies for the S12L set.^157^ Dimer centered basis sets with basis set extrapolation and a scaling factor were used to account for the absence of diffuse functions. This procedure is based on the convergence tests of the S22 dataset. However, dispersion terms were found to be overestimated by using a scaling factor for simple dispersion dominated systems like methane and ethane dimers. In a similar manner to Hesselmann and Korona, a scaling factor was used by Sharapa *et al.* to compute the C_60_ dimer interaction energy.^158^ A basis set without diffuse functions leads to an underestimated DFT-SAPT C_60_ dimer interaction energy curve.^158^ However, the use of such scaling factors for supramolecular systems is scarcely validated. We can see from Fig. 4 that DF-DFT-SAPT predicts contrasting trends for *π* − *π* stacked complexes: underbinding the 3a and 3b complexes by 4 kcal/mol and overbinding the 4a and 4b complexes by 7 kcal/mol. These discrepancies are larger than the estimated error from the back-corrections. It is not clear to what extent these discrepancies arise from basis set incompleteness or the amount of screening in dispersion that DF-DFT-SAPT is able to capture. The recent work of Lao and Herbert^123^ shows that XSAPT-DFT, a method that utilizes many-body polarized wavefunctions, also overbinds the 4a buckyball-catcher complex by ∼7 kcal/mol. By contrast, structures 5a to 6b, which are hydrogen bonded and cation-dipole complexes, are predicted within 1 kcal/mol by DF-DFT-SAPT.^157^

Calbo *et al.*^127^ used a more well-established method of obtaining basis set completeness: ΔDLPNO-CCSD(T) energies were corrected for basis set incompleteness by computing the CBS limit at the MP2 level.^159^ However, it has been shown that for systems containing long-range dispersion interactions, a tighter threshold is needed to capture long-range electronic correlation accurately. The ΔDLPNO-CCSD(T) approach was shown to yield a mean absolute error (MAE) of 1.1 kcal/mol, with respect to Emp-v1 for S12L and ΔQCISD(T) energies for L7. The calculations could not be converged for the buckyball catchers, 4a and 4b in the S12L, highlighting the numerical instabilities that are encountered for relatively modest basis set sizes when molecules contain a high degree of spatial orbital overlap. The ΔDLPNO-CCSD(T) error can be as much as 7.6 kcal/mol, for the cation-containing complex 7a. However, the back-correction itself is difficult to ascertain for this complex given that the implicit solvation model is particularly problematic for charged systems.^160^ Interestingly, the ΔDLPNO-CCSD(T) and MP2C methods disagree by ∼15 kcal/mol for complex 7a, which is the largest discrepancy between these two methods within the S12L dataset. All other differences between ΔDLPNO-CCSD(T) and MP2C surmount to less than 6 kcal/mol per complex. This indicates that even without back-corrected experimental data, there is a large inconsistency in the relative performance of these methods. There are a number of physical interactions that have to be accurately accounted for in order to attain chemical accuracy. For instance, the presence of the iron cation in 7a indicates that there may be a degree of degeneracy leading to static correlation. This form of correlation generally requires a multi-determinant wavefunction, which is not given by either ΔDLPNO-CCSD(T) or MP2C since both are based on a HF single determinant. Second, the dispersion interactions are long-range and it is not known to what extent dynamic correlation is accounted for in this system within the DLPNO formalism. Given that both Emp-v1 and DF-DFT-SAPT interaction energies for 7a are significantly larger than ΔDLPNO-CCSD(T), it is likely that the latter is underestimating the interaction energy.

Notably, many of the studies discussed compare their results to the DMC reported values from the work of Ambrosetti *et al.*^56^ The DMC interaction energies were computed for half of the S12L set, and a considerable amount of computational effort was conceded in obtaining stochastic error bars of less than 2 kcal/mol. Taking into consideration the stochastic error in the DMC results and the estimated error in the back-corrected experimental values, the DMC predictions agree within 2.2 kcal/mol for the structures computed, as can be seen from Table II. To establish if this remaining error stems from the empirical back-correction or the DMC prediction, the approximation for the DMC calculations should be considered. First, the effect of the fixed node approximation was tested by computing the interaction energy for the 2a complex with different trial wavefunctions. In this way, different nodal surfaces are used and any impact on the interaction energy should be noticeable. The interaction energies using two different trial wavefunctions were found to agree within statistical errors of 1.2 kcal/mol.

Second, the time step used in the calculation (0.002 in atomic units) was compared against a time step twice smaller. The resulting DMC interaction energy for the 2a complex was not affected (within the stochastic errors of 1.2 kcal/mol). However, the buckyball-catcher (4a) complex of S12L was recomputed with the improved DMC algorithm by Zen *et al.*^137^ The original DMC interaction energy from Ambrosetti *et al.*^56^ was found to be 4.6 kcal/mol underbinding. The improved algorithm brings the DMC interaction energy of the 4a complex into good agreement with Emp-v2. This indicates the presence of time step errors potentially in prior studies where system specific time step convergence was not undertaken and time steps were considered to be transferable between complexes of a dataset. The agreement between DMC (using the old algorithm) and Emp-v2 for non-dispersion bound complexes (see Fig. 4) suggests that the size-consistency error mostly affects complexes with a large contribution from dispersion interactions.

In the case of the cationic complex 6a from S12L, the DMC result is in excellent agreement with the back-corrected experimental values. Note that the first estimate of the back-corrected interaction energy for the 6a complex from Grimme^23^ is 5 kcal/mol less binding than the revised value by Sure and Grimme.^156^ The estimated error is 6 kcal/mol in Emp-v2, showing that the implicit solvation models used in estimating the interaction energies from experimental association constants are less reliable for charged systems. As such, DMC can be considered more robust for the interaction energy for the supramolecular complexes than the back-corrected values. Importantly, this statement can only be made due to the thorough convergence of the DMC calculations. Indeed, DMC is not considered a black-box method, but by executing careful convergence protocols, it has been shown to yield excellent results for large complexes.

On the whole, there is still a lot of scope for improving our *ab initio* predictions of supramolecular systems. We have seen that the high degree of anisotropy in the S12L dataset is difficult to establish with typical benchmark methods. Interactions consisting mostly of electrostatics can be predicted with greater accuracy than dispersion bound systems, and *π* − *π* stacking structures can be particularly challenging. However, major discrepancies can also be seen for the iron cation containing complex (7a). In the following class of systems, molecular crystals, we will consider systems with generally fewer symmetry reduced degrees of freedom, but extensive periodic arrangements.

## V. MOLECULAR CRYSTALS: A RICH LANDSCAPE WHERE EVERYTHING COUNTS

Small differences in interaction energies are known to affect various macroscopic properties, and this is critically the case in pharmaceuticals. Polymorphism, more specifically, is common in molecular crystals and refers to the existence of numerous meta-stable crystals for a given chemical compound. Polymorphs of the same molecule can have lattice energies within a few kJ/mol of each other, necessitating precise predictions and measurements. The lattice structure and energy of polymorphs impact on their solubility and, therefore, on their activity as drugs. Due to the important role that kinetics play alongside thermodynamics, experiments may produce a particular polymorph, only for another more stable polymorph to be discovered later.

There have been notable examples of pharmaceutical polymorphs being found later with costly outcomes. A well-known example is ritonavir which was initially distributed in oral capsules, containing form I polymorph of the molecule.^161^ Two years after, it entered the market in this form, and a more stable form II polymorph was discovered. Due to higher stability of form II ritonavir, it was much less effective in the capsule form and production had to be halted as a result.^37^ It is therefore imperative for experimentalists to be aware of the most stable polymorphs and the relative stabilities amongst them.

Experiments can determine the absolute sublimation enthalpy of molecular crystals to within 5 kJ/mol and provide the geometry of crystals from x-ray diffraction. However, it can be difficult to experimentally find different polymorphs without knowing *a priori* the lattice structures a molecule can crystallize into. To this end, force-field models or DFT methods can be used to predict possible polymorphs and their relative stabilities.^38,153,162,163^ Due to the scarcity of reference information for molecular crystals, the predictive power of *xc* functionals and force fields is not well established for organic crystals. Indeed, Brémond *et al.* assessed 59 functionals for the prediction of structural parameters in relatively small organic molecules and found a large spread of results.^163^

Two particular obstacles to computing reference lattice energies exist for molecular crystals. First is the use of periodic boundary conditions in high-level wavefunction based methods and second is the large size of the unit cells needed to model such systems accurately. The extensiveness of crystals requires periodic boundary conditions to be implemented, as is routinely done in DFT codes. However, fully periodic systems have been tackled with CCSD(T) for small unit cells consisting of a few atoms.^103,131,164–166^ Although this area is actively being developed,^164^ organic crystals are still too expensive to compute with periodic coupled cluster theory. As discussed in Sec. III, embedding and fragment approaches have been used with quantum-chemical methods for molecular crystals previously. On the other hand, the RPA and QMC are both readily used with periodic boundary conditions and lend themselves to predicting the energies of molecular crystals.

The predictive power of the high-level wavefunction based methods that we consider here is determined by comparison with the experiment. However, the non-zero finite temperatures at which experiments are conducted means that thermal effects and zero-point energies separate the experimental data from the theoretically obtained absolute energies. This gap is filled by back-correcting experimental sublimation enthalpies, yielding comparable lattice energies. It is important to note that the back-correction itself is computed from DFT or classical models and can therefore introduce uncertainty on top of the experimental values. Given that very small energy windows dictate the properties of molecular crystals, errors in the back-correction need to be minimized. This is non-trivial, and therefore, an alternative indicator of accuracy is cross-validation between several high-level wavefunction based methods.

**FIG. 5.**
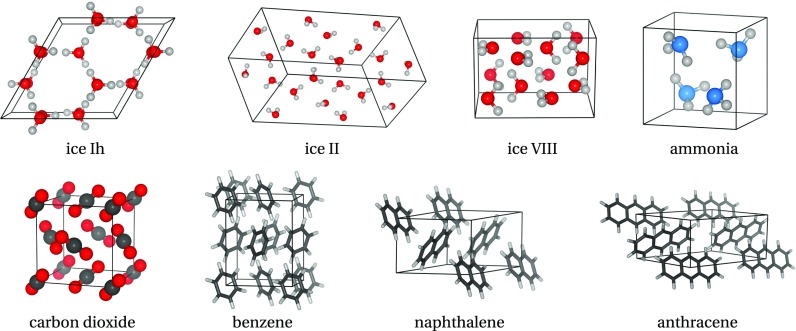
Molecular crystals from the work of Zen *et al.*^57^ The molecular solids include three ice structures (ice-Ih, ice-II, and ice-VIII), three *π* − *π* stacking structures (benzene, naphthalene, and anthracene), and ammonia and carbon dioxide crystals.

In the following, we report on the most accurate computations of molecular crystals from high-level wavefunction-based methods. First we address the efforts made for the more widely computed benzene crystal and compare the variety of methods applied to it. Second, we focus on the wider applicability of wavefunction based approaches, using a diverse set of molecular crystals shown in Fig. 5.

### A. The benzene crystal test

The benzene molecular crystal is one of the most studied systems and is a desirable test case; as benzene is a structurally rigid non-polar molecule with a polarizable charge density, its structure and stability are highly susceptible to dispersion interactions. In addition, its anisotropic polarizability can result in non-additive dispersion interactions,^5^ which requires going beyond the molecule-pairwise additive formalism. The experimental lattice energy of the benzene crystal is −50.6 kJ/mol, after back correcting for thermal effects and zero-point energy contributions.^57,167^ It should be noted that an uncertainty of a few kJ/mol can be expected in this value as a result of the extrapolation and back-correction used to obtain it. Approaches based on SAPT-DFT, CCSD(T), the RPA, and DMC have all been applied to the benzene crystal with varying success, as we presently discuss (Table III).

**TABLE III.** Lattice energy, *E*_*latt*_, in kJ/mol for the benzene crystal as reported in previous studies and the corresponding error with respect to the lattice energy from the back corrected experimental^57^ sublimation enthalpy, $\Delta H_{sub}^{exp}$.

Methods	*E*_*latt*_	$E_{latt}-BC\left[\Delta H_{sub}^{exp}\right]$
DMC^57^	−52.1 (0.4)	1.1
Emb. ΔCCSD(T)^109^	−51.2	−0.6
OSV-LCCSD(T)^168^	−54.6	−4
RPA^169^	−45.2	5.4
RPA+GWSE^169^	−51.5	−0.9
MP2^109^	−61.6	−11
Frag. ΔCCSD(T)+3B^170^	−51.5	−0.9
Frag. ΔCCSD(T)^110^	−56.4	−5.8
Frag. SAPT-DFT+3B^171^	−50.3	0.3
Frag. SAPT-DFT^172^	−58.4	−7.8
BC[$\Delta H_{sub}^{exp}$]	−50.6	

Podeszwa *et al.* used SAPT-DFT with the fragment method to compute the lattice energy of the benzene crystal at 0 K.^171^ The fragment approach that was employed computes the energy of dimers within the crystal and sums over 50 symmetrically distinct benzene dimer pairs, covering interaction distances up to 11 Å. The remaining energy of the crystal was computed by an asymptotic expansion using just under 9000 pairs. Based only on dimers, the SAPT-DFT predicted lattice energy of benzene is −57.2 kJ/mol. It was recognized that many-molecule effects or screening (e.g., the interaction of a dimer in the presence of other molecules) can contribute well beyond 1 kJ/mol to the lattice energy. As such, an attempt was made to recover three-molecule interactions by using MP2 to calculate the interaction energy of symmetrically distinct trimers within a 9 Å separation distance. The MP2 three-molecule energy contribution to the lattice energy was found to be 6.9 kJ/mol. The resulting lattice energy reported by Podeszwa *et al.*, using SAPT-DFT for dimers and MP2 for trimers, is −50.3 kJ/mol.^171^ This trimer corrected result is within 0.3 kJ/mol of the experimental benzene lattice energy. However, MP2 is known to overestimate *π* − *π* dispersion interactions. Therefore, the improvement in the MP2 corrected SAPT-DFT predicted lattice energy probably involves some compensation between the underestimation of dispersion from the dimer-only SAPT-DFT approach and the overestimation of *π* − *π* interactions from MP2. It follows that the use of MP2 to compute energy contributions to the lattice energy for molecules with *π* − *π* interactions, such as benzene, introduces further uncertainty and should be regarded with caution.

A fragment approach was also used by Ringer and Sherrill using ΔCCSD(T) to compute dimer energies.^110^ Trimer energies were neglected in that study under the assumption that they do not contribute significantly to the lattice energy. The fragment ΔCCSD(T) approach predicted a benzene crystal lattice energy of −56.4 kJ/mol. Note that this is within 0.8 kJ/mol agreement with the dimer-only SAPT-DFT prediction, indicating that SAPT-DFT is accurate for considering the interaction of benzene dimers. Nonetheless, it is evident that beyond two-molecule interaction energies are required for the accurate prediction of the benzene crystal—preferably from a theory that does not overestimate dispersion interactions. It is also important to emphasize that a lattice energy difference of a few kJ/mol is large enough to have drastic effects on macroscopic properties. Later, Sherrill and co-workers computed the ΔCCSD(T) interaction energy for trimers in the benzene crystal and showed that it contributes 3.7 kJ/mol (about 7%) to the lattice energy.^170^ The trimer corrected ΔCCSD(T) lattice energy for benzene is −51.5 kJ/mol.

The successive improvement of the results from fragment based quantum chemistry approaches demonstrates a simple drawback: without an already known reference energy, it is difficult to know how many clusters to include. One can compute many-molecule contributions (e.g., trimers, tetramers, pentamers, etc.) more straightforwardly with methods such as localized CCSD(T) approaches. Reference results for larger clusters can be used to establish the convergence behavior of the fragment approach. Ultimately, however, a fully periodic framework is the ideal method for computing lattice energies.

To efficiently account for periodicity whilst retaining a degree of accuracy from high-level methods, an embedding or quantum mechanics/molecular mechanics (QM/MM) approach can be applied. QM/MM encompasses a number of methods that couple high-level wavefunction-based methods with less costly mean-field or classical models. High-level theory is applied to treat the more complex or strongly interacting part of the system, whilst a mean field approach such as DFT can be used to compute the remaining components of the system. Wen and Beran used ΔCCSD(T) and a carefully parameterized force field to compute the periodic lattice energy of benzene, along with a few other molecular crystals.^109^ They obtained a benzene lattice energy of −51.2 kJ/mol, which is in the same range as the lattice energies predicted by the ΔCCSD(T) and SAPT-DFT fragment approaches. Interestingly, the partitioning between ΔCCSD(T) and the force field was based on the components of the energy as opposed to a purely spatial partitioning. The reliability of this method is coupled to the accuracy of the force field which contributes a significant proportion of the benzene lattice energy. However, the incomplete quantum mechanical treatment results in the neglect of long-range and higher-order dispersion interactions. To alleviate this somewhat, a semi-empirically determined Axilrod-Teller-Muto (ATM) term was included in the force-field, in order to approximate the contribution from three-body interactions. Although the ATM term accounts for some portion of the missing interactions in the force field, its accuracy is also not guaranteed.

Recall that there is a strong motivation for computing accurate lattice energies with enough resolution to distinguish polymorphs. With that in mind, the goal is to compute long-range interactions with quantum mechanical accuracy. To this end, the random phase approximation (RPA) was used by Lu *et al.* RPA benzene lattice energies, −44 and −47 kJ/mol, were computed for Kohn-Sham (KS) orbitals using the local density approximation (LDA) and a generalized gradient approximation (GGA), respectively.^173^ Note the impact of the approximate KS orbitals on the RPA lattice energy is already a few kJ/mol. Del Ben *et al.*^166^ similarly found a difference of ∼8 kJ/mol in the predicted lattice energy of the benzene crystal, between RPA based on HF and PBE orbitals. In addition, in comparison to previously discussed quantum chemical results, the RPA estimates a weaker interaction in the lattice. This is typically seen for RPA predictions of dispersion dominated interactions and stems from the lack of single excitations, although higher-order exchange terms are also missing. In short, RPA lends itself very well to the computation of periodic systems with dispersion, but it is essential to include further terms for completion. The need for further terms has been demonstrated in a number of studies, and promisingly, their inclusion is becoming more routine. A more recent RPA+GWSE prediction of the benzene crystal lattice energy by Klimeš is −51.5 kJ/mol.^169^ Clearly, the GW singles excitations (GWSE) contribution is significant and the final result is within 1.3 kJ/mol of the back-corrected experimental lattice energy of −50.2 kJ/mol. The RPA+GWSE method requires careful convergence of calculation parameters (e.g., basis set size and finite size effects), and the effect of SOSEX could be considered further. However, one can be satisfied that correlation is treated seamlessly for a molecular crystal within the RPA+GWSE method.

In addition to the RPA+GWSE method, Zen *et al.* demonstrated the power of QMC for establishing the lattice energy of crystals.^57^ In the case of the benzene crystal, DMC predicts −52.1 ± 0.4 kJ/mol for the lattice energy. The DMC lattice energy is well within the predicted lattice energies of the RPA+GWSE and quantum chemistry methods. The consensus of several high-level methods provides confidence in the benzene lattice energy—even more than on the computed back-correction that is applied to the experimental measurement. In Sec. V B, we consider a wider set of molecular crystals: ice-Ih, ice-II, ice-VIII, CO_2_, NH_3_, naphthalene, and anthracene.

### B. Molecular crystals of all shapes and sizes

**FIG. 6.**
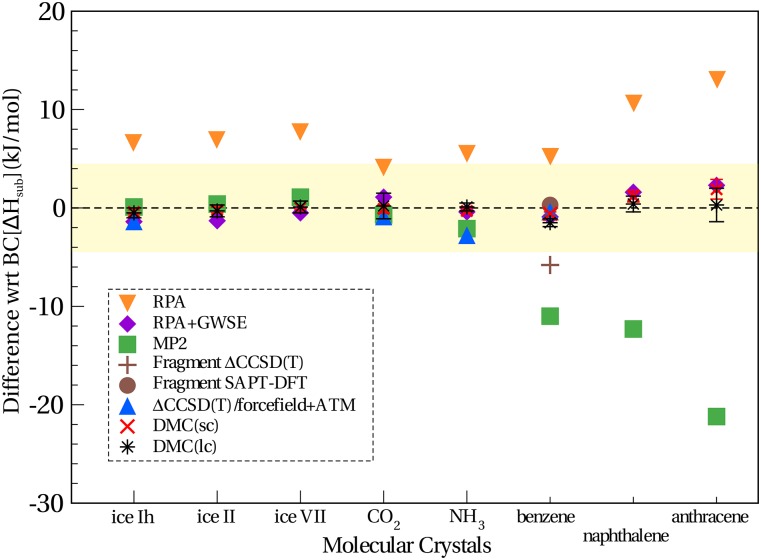
Differences in the interaction energy between a number of high-level wavefunction based methods and the revised back-corrected experimental sublimation enthalpies (BCΔ*H*_*sub*_). The chemical accuracy of ∼4.2 kJ/mol is indicated by the yellow shaded bar. Data are adapted from previous studies.^47,57,108,109,168,169,177^

Whilst benzene is the *de facto* test case for molecular crystals, a more representative dataset of molecular crystals is needed to thoroughly test the application of methods. For this purpose, the C21^147^ and the later refined X23^148^ datasets are particularly well suited. These datasets include a variety of molecules that are found in nature and are common in chemical synthesis. Zen *et al.* established QMC lattice energies for a subset of the C21 molecular crystal dataset and three ice polymorphs additionally.^57^ The two methods of DMC, DMC(lc) and DMC(sc), shown in Fig. 6 and Table IV, refer to DMC computed in large cells (lc) and small cells (sc). In other words, finite size effects are either prevented in the brute force approach by DMC(lc) or, at an order of magnitude smaller computational cost, in DMC(sc) using smaller cells and a correction to finite size effects (i.e., model periodic Coulomb interaction).^57,174–176^ The computed crystals comprise a reasonably diverse range of molecules with lattice energies ranging from 28 to 105 kJ/mol, accompanied by DMC stochastic errors from 0.1 to 1.7 kJ/mol. In addition to obtaining lattice energies in close agreement with back-corrected experiments consistently across hydrogen-bonded, dispersion dominated, and mixed bonded crystals as can be seen in Fig. 6, the relative stabilities of ice polymorphs were also correctly predicted. This is a significant feat since the energy difference between the ice polymorphs considered in the study is less than 2 kJ/mol and well under the typically targeted chemical accuracy of ∼4.2 kJ/mol.

**TABLE IV.** Lattice energies, *E*_*latt*_, and mean absolute errors (MAEs) in kJ/mol for a small variety of molecular crystals, predicted in previous studies. The last column reports the lattice energies from back corrected (BC) experimental sublimation enthalpies ($\Delta H_{sub}^{exp}$). The stochastic errors for the DMC results are reported in parentheses. DMC(lc) and DMC(sc) refer to DMC calculations performed in large and small supercells, respectively. The MAEs for DMC take into account the stochastic errors, but for comparison, the parentheses show the MAE without this.

Molecular crystal	DMC(lc)^57^	DMC(sc)^57^	Emb. ΔCCSD(T)	RPA^169^	RPA+GWSE^169^	MP2	BC[Δ*H*_*sub*_]
Ice Ih	−59.3 (0.5)	−59.2 (0.2)	−58^47^	−52	−60.2	−58.7^47^	−58.8
Ice II	−59.1 (0.6)	−59.0 (0.3)	−58^47^	−51.7	−60.1	−58.4^47^	−58.8
Ice VIII	−57.3 (0.6)	−57.4 (0.1)	−55.4^47^	−49.5	−57.9	−56.3^47^	−57.4
Carbon dioxide	−28.2 (1.3)	−28.5 (0.4)	−29.5,^109^ –28.1^108^	−24.1	−27.3	−29.1,^109^ –27.2^108^	−28.4
Ammonia	−37.1 (0.4)	−37.5 (0.1)	−40.2^109^	−31.5	−37.6	−39.3^109^	−37.2
Benzene	−52.1 (0.4)	−51.2 (0.2)	−51.2^109^	−45.2	−51.5	−61.6^109^	−50.6
Naphthalene	−78.8 (0.8)	−78.0 (0.6)	n.c.	−68.4	−77.6	−91.5^177^	−79.2
Anthracene	−105.5 (1.7)	−103.9 (1.0)	n.c.	−92.6	−103.5	−127^177^	−105.8
MAE	0.2 (0.4)	0.3 (0.6)	1.4	7.7	1.2	6.1	

In light of the scarcity of reference information, demonstrating full agreement between high-level methods originating from different theories and frameworks is compelling and it is a necessary step towards establishing the reliability of different methods. Such a study requires a concerted effort from researchers, as has been demonstrated for the interaction of water on h-BN^13^ and LiH surfaces.^107^ Zen *et al.* show that such an agreement can be achieved within 2 kJ/mol using DMC, the RPA+GWSE, and carefully embedded ΔCCSD(T). This comparison also highlights which methods are more suitable to predicting molecular crystal lattice energies, namely, DMC and the RPA+GWSE. Both of these methods can be applied seamlessly to periodic systems, and provided that theoretically motivated steps (as opposed to empirical fitting) are taken to ensure fully converged results, excellent accuracy can be achieved. Although the same can be said for embedded ΔCCSD(T) approaches, due to the different theoretical bases of the composite methods, the non-covalent interactions are not treated on the same balanced footing.

By making cross-comparisons between methods and understanding the level of approximations made in each case, it is clear that two main factors should be satisfied to achieve the best accuracy. First, a many-body approach to correlation (or dispersion) is needed in order to compute molecular crystals with sufficient accuracy to distinguish polymorphs. This implies that periodic or embedded QM/MM approaches are preferred to fragment based approaches. Second, the non-covalent interactions should be treated on a balanced footing, which favors the use of a single method such as DMC or the RPA+GWSE as opposed to the composite QM/MM methods. However, when it is known that the long-range interactions in a system are not important, a QM/MM method is likely to be useful.

## VI. THE DFT WORKHORSE ON A COMPLEX MOLECULAR TRACK

Amongst first principles methods, DFT-based predictions are overwhelmingly relied upon for quantum-mechanical predictions of organic crystals as well as other materials. Indeed, in the organic crystal blind tests organized by the Cambridge Crystallographic Data Centre, the leading approaches for the prediction of polymorph stabilities employ vdW-inclusive DFT methods.^162^ In general, however, DFT-based approximations suffer from the lack of a systematic route to improved accuracy due to the unknown form of the exact *xc* functional. Countless approximations for the *xc* functional of different flavors have been developed in light of this, some of which include approximations for vdW interactions. Several studies have shown that such vdW inclusive functionals predict a wide spread of interaction energies for beyond modestly sized non-covalently interacting systems.^14,15,47,69,123,147,178–180^

A number of vdW-inclusive functionals account for pairwise interactions between charge densities (e.g., atoms and molecules) and therefore neglect higher-order interactions,^62,63^ which are particularly important for non-additive dispersion interactions. In addition, several such vdW-inclusive *xc* functionals treat short and long-range correlation separately since the former is well approximated with the underlying GGA formalism. Thus, further approximations are needed to interface short and long-range correlation. The method of interfacing the short and long-range correlation in an *xc* functional can particularly impact on interactions at 3-7 Å separation distances.^181,182^ Indeed, recent studies have shown that the incorrect behavior in the medium-range distance of electron correlation can lead to an overestimation of the adsorption energy of molecules in low-dimensional materials, such as a carbon nanotube.^13^ This is likely to be particularly noticeable in systems such as molecular crystals and supramolecular systems, where a considerable amount of dispersion is expected to fall in this medium-range of interaction. Given the continued usefulness of DFT, let us briefly comment on recent advances in the use of DFT based methods for supramolecular complexes in Sec. VI A, followed by molecular crystals in Sec. VI B.

### A. Supramolecular systems with vdW inclusive DFT

The availability of high-level reference information for the S12L dataset, particularly from back-corrected experiments^23,156^ and later DMC,^56^ spurred several studies to provide performance tests for vdW inclusive *xc* functionals.^72,73,127,152,156^ Interestingly, the series of studies undertaken by different groups resulted in the refinement or clarification of the parameters related to some of the dispersion approximations used in DFT, which we will recount here. Such discussions and findings are crucial for the standardized and reliable use of DFT approaches and exemplify the usefulness of accurate reference information.

Let us begin with the work of Risthaus and Grimme that undertook important benchmarking,^72^ comparing the interaction energies from D2, D3, dDsC, VV10, vdW-TS, and exchange-hole dipole moment (XDM) based DFT approaches and also the highly parameterized M06-L functional. Grimme’s D2 in combination with PBE was found to produce the smallest mean absolute error (MAE) for the S12L dataset (1.5 kcal/mol) in that study, but as the authors also remark, the good performance of PBE+D2 can be attributed to error cancellations. This is indicated by their finding that the D3 approach which approximates three-atom dispersion appeared to be less accurate than PBE+D2, with an MAE of more than 2 kcal/mol. The absolute difference in the MAE does not appear significant, but when employing first principles methods, a desirable feature is to see increased accuracy as more physics is accounted for. Indeed, the PBE+vdW-TS functional, based on a pairwise atom-atom description, was found to consistently overbind the host-guest complexes of the S12L dataset, resulting in a larger MAE of 5.3 kcal/mol. In comparison to the D2 method, the overestimation of interaction energies by the vdW-TS method is physically expected due to the neglect of beyond atom-pairwise dispersion in its parameterization. Further evidence for error cancellation in the D2 method was demonstrated by Ambrosetti *et al.* in their decomposition of the many-body dispersion (MBD) energy.^56^ The MBD framework provides an approximation to the RPA correlation energy via atom centered quantum harmonic oscillators, and in this way, it accounts for beyond atom-pairwise dispersion. In the MBD decomposition of the interaction energy for a subset of the S12L dataset, truncation at the atom-pairwise level was found to result in an overestimation of 7%-13% in the dispersion component of the interaction energies. In fact, the PBE+MBD functional was found to yield a competitive MAE of 1.6 kcal/mol for S12L.

In addition to confirming the importance of beyond atom-pairwise interactions in large supramolecular complexes, benchmarking also revealed the need for the careful use of parameters for dispersion models that supplement *xc* functionals. For example, Risthaus and Grimme reported an MAE of more than 5 kcal/mol^72^ for the XDM model of Johnson, but as Otero-de-la-Roza and Johnson later showed,^152^ this large MAE was caused by shortfalls in the convergence of the basis set as well as the wrong parameters being used in combination with PBE. Otero-de-la-Roza and Johnson investigated the effect of using different parameters in their damping function and remarked that with a reduced basis set error and optimized damping function parameters, the PBE+XDM MAE for the S12L dataset is in fact 1.2 kcal/mol^152^ when compared with the DMC results of Ambrosetti *et al.*^56^

Benchmarking efforts were also undertaken by Calbo *et al.* for double-hybrid density functionals combined with the VV10 long-range correlation of Vydrov and Van Voorhis.^127^ In their study, ΔDLPNO-CCSD(T) references were used for both the S12L and L7 datasets, and with this, they found MAEs of at least 2.5 kcal/mol. It would be interesting to see whether the MAE would be lower if DMC values are taken as reference. In addition, the study indicated that the L7 dataset is more difficult to predict accurately from DFT based methods. In particular, the increase in the number of hydrogen bonds in the L7 dataset could be challenging for DFT approximations.^154^ This is demonstrated in the recent work of Claudot *et al.*^69^ where the L7 dataset was benchmarked with several vdW inclusive *xc* functionals and significant discrepancies were found for hydrogen-bonded complexes. It has previously been shown, for example, that predicting the electrostatic interactions can require a more accurate description of Pauli exchange-repulsion,^70,154^ and therefore in the realm of DFT *xc* functionals, more expensive hybrid functionals might be needed to accurately compute the L7 dataset. However, the L7 reference data should be further revised in future,^123^ especially using a better converged basis set and more complete theory. This would be helpful, for example, towards settling the performance of the DFT *xc* functionals in large finite molecular systems.

### B. DFT for molecular crystals

The C21 and X23 datasets have been profoundly useful for comparing vdW approximations in DFT methods and highlighting the benefits and shortfalls of different approaches. Compared to supramolecular systems, these molecular crystals have smaller theoretical back-corrections due to the molecules being more rigid, and the geometries are better established, thanks to low-temperature diffraction data. As such, C21 and X23 can be used by force field methods alike, helping further development in classical as well as quantum-mechanical approaches.^183,184^

Dispersion inclusive *xc* functionals have been benchmarked in previous studies and some reviews elaborate in more detail on the performances.^62,169^ Generally, vdW-inclusive methods have MAEs up to a few kcal/mol of the back-corrected experimental lattice energies. However, as already discussed, the lattice energy of molecular polymorphs can be less than that energy window. Therefore, predicting the correct ranking of polymorphs requires accuracy in a small energy window as well as the inclusion of entropic effects (which we discuss later). Interestingly, it has been found that invoking beyond two-body interactions, as in the MBD model, lowers the MAE to 2 kcal/mol and improves the accuracy of polymorph ranking.^22^ Indeed, the MAEs of PBE0+MBD (0.9 kcal/mol), PBE+MBD (1.4 kcal/mol), and PBE+D3 with the three-atom ATM term included (1.1 kcal/mol) for the X23 organic crystal dataset demonstrate the remarkable accuracy that can be achieved for the lattice energies.^22,72,185^ Moreover, comparisons between the TS-vdW and MBD methods can provide useful physical insight on the dispersion interactions of a dataset. For example, many-atom interactions in the MBD improve the MAEs of molecular datasets, such as the X23, by 2-3 times, when coupled with the same underlying *xc* functionals.^22^ The impact of three-atom dispersion terms can also be seen with the D3 method with and without the ATM term for the X23 dataset.^185^ Evidently, non-additive dispersion plays an important role in the long-range interaction of molecular crystals.^186^

Of the dispersion inclusive *xc* functionals that are effectively atom-pairwise only, the generally named vdW-DFs overestimate the lattice energies of the X23 dataset by ∼10%.^75^ Meanwhile, the XDM model has an MAE of 1 kcal/mol when combined with the B86b functional.^75^ The XDM model contains higher-order terms in the dispersion expansion of the atom-pairwise regime, accounting for interactions that decay as *R*^−8^ and *R*^−10^, for inter-atomic separation *R*. Although the XDM model does not account for beyond atom-pairwise non-additive dispersion interactions, the inclusion of higher-order terms within the atom-pairwise formalism in the XDM approach reduces the overbinding that otherwise results from using only the *R*^−6^ term in this approach. Overall, some of the physically motivated and less empirical DFT+vdW methods can be considered rather promising models for molecular crystals, which can be further developed towards better accuracy.

## VII. DISCUSSION

To date, predictions of small molecular systems and molecular materials have seen significant improvements. State-of-the-art computational methods, such as CCSD(T) and DMC, are able to predict interaction energies for symmetric systems with remarkable accuracy. Yet at the intersection of these systems, molecular crystals and supramolecular complexes are challenging target systems, which exhibit a complex interplay of non-covalent interactions. Here, we first present a succinct general framework in Sec. VII A for assessing the quality of the reference information computed from *ab initio* methods. Second, we briefly discuss the role of non-equilibrium effects on the context of beyond modestly sized molecular systems in Sec. VII B.

### A. Criteria for benchmark accuracy predictions

We have seen that wavefunction-based methods are not consistently in agreement due to the different levels of treatment for physical interactions such as dispersion. The neglect of higher-order terms in perturbation theories or practical approximations, for example, in the basis set, strongly delineates the findings. When navigating the literature for reference information, it is important to keep in mind the pitfalls of the various so-called benchmark methods. Table I in Sec. III gave an overview of the typical wavefunction based methods that are used to compute benchmarks. Here, we suggest the following criteria as a checklist for obtaining a sense of the quality of the reference:

• Reproducibility: this not only concerning experiments but computational methods also. As DFT studies demonstrate,^187^ it is not given that different codes, implementations, and basis set approximations yield the same result for a particular theory.
• Systematic convergence: computational methods rely on numerical approximations in practice, but it is important that such approximations can be systematically tested and converged. For example, basis set size and finite size errors should be thoroughly tested. To this end, the use of *ad hoc* scaling factors should be avoided.
• Theoretical completeness: this is what currently separates most DFT based methods^178^ from the so-called high-level wavefunction based methods. More specifically, the understanding of the interaction terms that are included in a theoretical framework underpins to what extent a method can provide insight and reliability.
• Consensus: a compelling trait of accuracy is the agreement between methods of different theoretical bases, for example, where possible CCSD(T), DMC, and the RPA+GWSE should be shown to predict the same interaction energies. Such efforts are being made more recently^13,57^ and continue to provide the necessary grounding for these reference methods.

Following our prescribed framework above, the most accurate computational work for non-covalently bonded large systems is given by Zen *et al.*^57^ for moleular crystals. Overall, DMC can provide excellent reference information for molecular crystals and large supramolecular complexes. As DMC is not considered a black-box method, it requires a great deal of effort to ensure that all numerical approximations in the calculation are not creating a bias in the result. In addition, the computational cost of DMC depends largely on the size of the target stochastic error, but as shown by Zen *et al.*, developments in the algorithm have resulted in significant savings. Second, the RPA+GWSE method has been shown to predict interaction energies in agreement with DMC, and as an analytic approach, it does not involve statistical errors. As such, reliable results can be expected from the RPA+GWSE for non-covalently interacting systems. One bottleneck for the RPA+GWSE appears to be the memory requirements that increase substantially with the basis set and system size. However, it is not entirely prohibitive since accurate RPA+GWSE computations for unit cells with over 300 electrons have been done, for example, in the water adsorption study of Al-Hamdani *et al.*^188^

Quantum-chemical methods, such as DLPNO-CCSD(T), are useful approximations to canonical CCSD(T), but drawbacks include slow basis set convergence and the difficulty of applying it to periodic systems. It would also be beneficial to study the effectiveness of DLPNO-CCSD(T) for highly delocalized systems, where the dispersion interactions are particularly long-ranged. Finally, SAPT-DFT methods continue to provide useful physical insight for interacting dimers and potentially more reliable results than vdW inclusive of DFT approximations.^150^ Dimer systems remain the main scope of SAPT, thus limiting its application to molecular materials. As larger dimer complexes are increasingly studied, it remains to be seen whether the level of perturbation theory within DFT-SAPT is sufficient to describe the necessary interaction terms needed to describe the dispersion interactions in such systems.

### B. Non-equilibrium effects in beyond modestly sized molecular systems

In this perspective, we have focused on the currently available information from high-level methods for non-covalently bonded systems, but beyond 0 K interaction energies, non-equilibrium effects are fundamental in the properties and application of such systems. In particular, thermal and anharmonic effects feed into the back-correction from experiments and therefore comprise the effects that separate high-level theoretical predictions from experimental measurements. Furthermore, the vibrational and zero-point effects play a major role in the free energy stabilities of polymorphs in organic crystals, as well as in the mechanical and elastic properties of molecular complexes. Well-established examples include aspirin, which in form I is entropically favored to form II. This has been rationalized by the non-additive many-atom interactions softening the low-frequency vibrational modes and stabilizing form I.^22^ It would be a significant step forward to be able to routinely predict these effects accurately.^189^ Particularly for anharmonicity, several methods are being actively developed to increase our understanding and knowledge. However, the main limitation is the cost of such calculations. Moreover, present implementations of stochastic methods like DMC do not lend themselves well to the calculation of dynamic contributions due to the additional computational cost required to compute gradients stochastically, as opposed to computing analytical gradients.

More practicable methods, i.e., DFT based formalisms, are needed to provide sufficient information to bridge theoretical computations with experimental measurements. Further benchmarking is needed to establish the reliability of DFT approximations for thermal effects. To this end, significantly more reference information is needed on non-equilibrium structures of large molecular complexes. The S66 × 8 dataset is an example of efforts to move towards describing small non-equilibrium dimers. Indeed, the accurate prediction of the interaction energy curve for water on hexagonal boron nitride resulted in a concerted effort in the electronic structure community to cement the agreement amongst high-level wavefunction based methods^13^ and provided useful information for force field development. In general, reference methods can be applied to organic crystals to compute the equation of state, as demonstrated by Zen *et al.*^57^ In addition, low-cost molecular dynamics simulations can be used to provide non-equilibrium configurations of complex systems, for which the interaction energies can be computed at the reference level. Producing reliable benchmark energies for non-equilibrium structures is a promising step forward in improving the accuracy of *xc* functionals and force field methods. This will be particularly useful, and as such, workhorse methods are likely to remain as the most efficient computing approaches.

## VIII. CONCLUDING REMARKS

Considering that experimental interaction energies are mostly available within the small-molecules regime (see Fig. 1), the current challenge is to apply computational methods in systems where there are scarce experimental data to guide us. If this can be achieved, we can consider accurately predicting macroscopic systems in future from first principles and, in doing so, gaining the ability to potentially explain highly complex phenomena. This involves clearly defined theoretical frameworks, which enable us to model physical interactions and to know which interactions are not accounted for. In other words, we can only obtain accurate predictions in unfamiliar systems by understanding the theoretical models that we apply, as well as their limitations. Indeed, significant strides have been made in the modeling of non-covalent interactions, particularly the application of wavefunction-based methods to periodic molecular crystals and large supramolecular complexes involving significant anisotropic and long-range dispersion energy contributions.

Molecular crystals and supramolecular complexes have typically been set aside in the development of computational methods. This is partly due to the lack of reference information in the past and the enormous complexity of these systems. However, establishing the reliability of various computational methods, such as *xc* functionals and force fields, means that such systems cannot be neglected. Whilst much progress has been made from the understanding of small dimers and solid-state matter, the greater challenge now lies in accurately predicting the molecular complexes that embody complex non-covalent interactions over particularly long distances.

A great deal of progress has been made, thanks to the developments by several theoretical groups, resulting in a steadily increasing pool of benchmarks for interaction energies of increasingly larger and complex molecules. Methods such as DMC, the RPA+GWSE, and DLPNO-CCSD(T) have reached the stage where they can be applied to systems consisting of up to hundreds of atoms. An issue that affects the performance of these methods is sometimes in the execution since it is tempting to take methodological short-cuts in the hope of providing much needed references. As outlined, there is scope for removing the uncertainties related to these methods, possibly by undertaking exhaustive protocols to ensure the reliability of the results. Upon consideration of the most pertinent reference studies for molecular crystals and supramolecular complexes, DMC can be expected to provide excellent benchmarks especially for systems where dispersion interactions in particular and correlation energy in general are not well understood. Still, it is important to keep in mind the effect of the fixed-node approximation in DMC interaction energies.

The RPA+GWSE method provides another consistent approach to establishing reference information, but missing terms such as the SOSEX could affect the results. In DMC and the RPA methods, the convergence of the energies is a formidable task with respect to unit cell sizes. In this case, various developments are being undertaken to introduce corrections for finite-size errors. For the approximate CCSD(T) type methods, a great deal of computational savings has been achieved at the cost of rigorous accuracy. Thus it is important to thoroughly test the limits of methods such as the DLPNO-CCSD(T).

An important concern within all of the aforementioned methods is the inclusion of static correlation. Whilst this is thought to be less important in the case of non-covalent interactions considered in this perspective, there can be systems where electronic near-degeneracies play a role in the interaction. At this time, the DMC framework provides a straightforward path to including this, as multiple determinants can be used in the trial wavefunction. However, it remains theoretically challenging to account for these effects via the RPA or CCSD(T) type methods. Another method, full configuration interaction QMC or FCIQMC for short, is therefore likely to provide an increasingly useful route to attaining highly accurate predictions. Unfortunately, in the case of molecular crystals and supramolecular systems, this method remains infeasible.

Finally, it is reassuring that high-level reference methods are capable of tackling challenging non-covalent complexes. To gain agreement with experiment however, non-equilibrium effects form an important challenge to overcome. Further developments to build on the ground state frameworks and extend them by accounting for thermal and anharmonic effects will cement the use of computational methods in making reliable predictions.
